# Extraocular Muscle Enlargement in Thyroid Eye Disease Using Volumetric Analysis

**DOI:** 10.7759/cureus.63843

**Published:** 2024-07-04

**Authors:** Kristen Park, Joy Li, Joyce Wen, Shirley Li, Jonathan Lee, Kayla Danesh, Nicolas Malkoff, Kimberly Gokoffski, Alexander Lerner, Vishal Patel, Sandy Zhang-Nunes, Jessica Chang

**Affiliations:** 1 Ophthalmology, Keck School of Medicine, University of Southern California, Los Angeles, USA; 2 University of Southern California (USC) Roski Eye Institute, Keck School Medicine, University of Southern California, Los Angeles, USA; 3 Radiology, Keck School of Medicine, University of Southern California, Los Angeles, USA; 4 Radiology, Mayo Clinic, Jacksonville, USA

**Keywords:** inferior rectus muscle, extraocular muscle enlargement, thyroid eye disease, superior rectus complex, extraocular muscle

## Abstract

Background and objectives: It is commonly taught that thyroid eye disease (TED) causes enlargement of the extraocular muscles (EOMs) in the following descending order: inferior rectus (IR), medial rectus (MR), superior rectus (SR), lateral rectus (LR), superior oblique (SO) and inferior oblique (IO). However, with recent literature challenging this notion, we aimed to compare EOM volumes in our cohort of TED patients.

Methods: We conducted a retrospective, non-randomized case-control study. Twenty-eight orbits from 28 unique patients with TED who had high-resolution CT scans were compared to 31 normal orbits, all from a single academic institution. Orbital soft tissues were manually segmented using ITK-SNAP 3.8.0 (http://itksnap.org), and soft tissue volumes of the control and TED orbits were compared using independent-sample t-tests.

Results: Of the TED orbits, 54% of SR/levator palpebrae superioris complex volumes (SRC) and 50% of IR volumes were greater than two standard deviations above the normal orbit average. Compared to controls, the mean SRC volume in TED subjects was 2.3 times enlarged, followed by the IR (2.1 times), SO (1.8 times), MR (1.7 times), LR (1.6 times), IO (1.6 times), and orbital fat (1.4 times) (*p* < 0.01 for all).

Conclusions: Our findings suggest that contrary to previous teaching, the SRC may be the most severely affected in TED.

## Introduction

Thyroid eye disease (TED) affects nearly half of patients with Graves’ disease [1]. Some of the most common manifestations of TED include upper eyelid retraction, edema, periorbital tissue and conjunctival erythema, and proptosis [1]. Another hallmark of TED is extraocular muscle (EOM) and adipose enlargement, with one study of 116 TED patients demonstrating that 90% had evidence of EOM enlargement on imaging [2]. The phenomenon is variable and unpredictable, and the degree of enlargement does not necessarily correlate with clinical disease severity or laboratory tests. It can affect the eyes asymmetrically and can preferentially affect either the fat or the EOMs [1,2]. There are a number of adverse effects that may result from the enlargement, including diplopia, proptosis, and compressive optic neuropathy. 

It is commonly taught that the order of EOMs affected in TED is as follows: inferior rectus (IR), medial rectus (MR), superior rectus (SR), lateral rectus (LR), superior oblique (SO), and inferior oblique (IO), with the IR and MR muscles being especially impacted [1-4]. However, the studies describing this pattern used less rigorous methodologies, and their findings have been challenged by more recent literature demonstrating the levator palpebrae superiors and SO muscles to be particularly affected in patients with TED [5-8]. Therefore, our study aims to examine EOM involvement in a clinic-based TED population using volumetric and maximum diameter-based measurements.

This article was previously presented as an electronic poster at the 2023 American Academy of Ophthalmology Meeting on November 3-6, 2023, and as a poster presentation at the North American Neuro-Ophthalmology Society (NANOS) 49th Annual Meeting on March 14, 2023.

## Materials and methods

Study design

A retrospective, non-randomized case-control study was performed. All CT scans included in the study were obtained according to the following parameters: helical mode, 0.65 pitch, 120 kVp tube voltage, dynamically modulated tube current, soft tissue kernel, 0.5 mm slice thickness, 200 mm diameter, 512 x 512 matrix.

Ethical considerations

This retrospective study adhered to the tenets of the Declaration of Helsinki and was approved by the institutional review board (IRB) of the University of Southern California, Los Angeles, CA (approval number: HS-20-00911).

Study criteria

For the TED group, 28 orbits from 28 patients who had been diagnosed with TED clinically by ophthalmologic evaluation and had received a CT scan between September 2014 and June 2021 were included. For each patient, the orbit that was determined to have worse clinical severity (based on clinical activity score, dysmotility, proptosis, lagophthalmos, eyelid retraction, or optic neuropathy) was selected for inclusion. Orbits from patients who had either undergone orbital decompression surgery prior to the CT scan date or who did not have high-resolution CT scans, such as patients with mild disease without imaging and patients with other imaging modalities such as MRI, were excluded. Thirty-one orbits from patients with no orbital pathology served as the control group for this study.

Procedure

**Figure 1:**
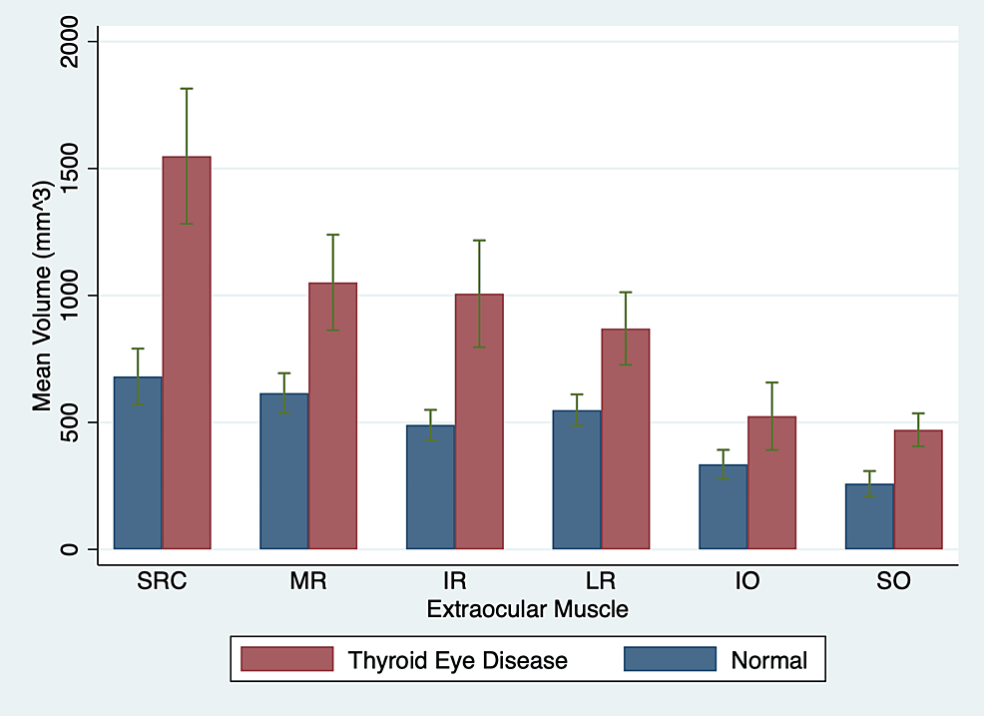
Average extraocular muscle volumes of normal orbits and thyroid eye disease orbits SRC: superior rectus complex; MR: medial rectus; IR: inferior rectus; LR: lateral rectus; IO: inferior oblique; SO: superior oblique

**Figure 2:**
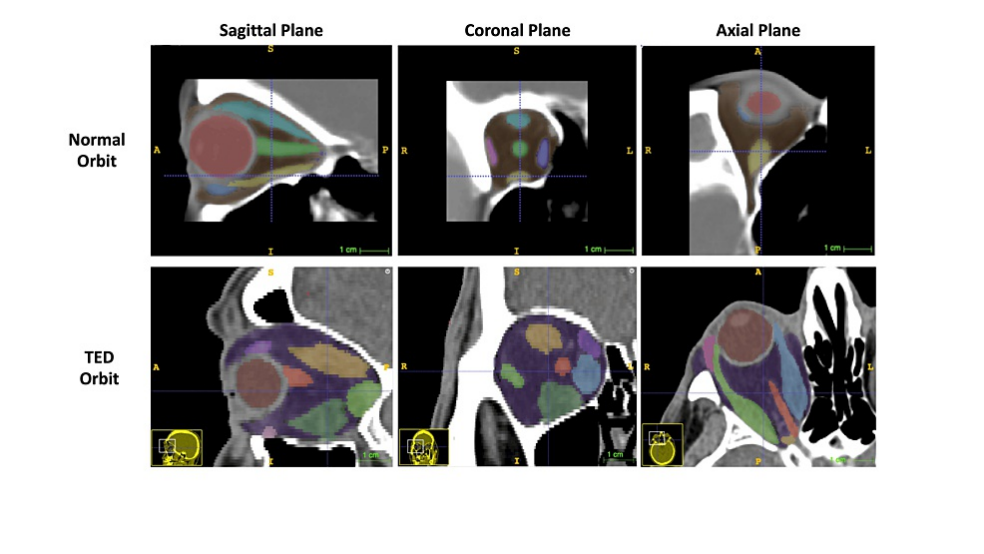
Display of a normal orbit and thyroid eye disease orbits on ITK-SNAP, demonstrating soft tissue enlargement in thyroid eye disease

The CT scans were manually segmented using ITK-SNAP 3.8.0 (http://itksnap.org), with the total volumes calculated from the segmentation areas of each CT slice (Figure 1). The superior rectus and levator palpebrae superioris were combined into the superior rectus complex (SRC) as they are not easily distinguishable on CT imaging [7, 9-12]. For each orbit scan, volumes of the orbital fat, SRC, LR, IR, MR, SO, and IO muscles were obtained (Figure 2).

Assessments

**Figure 3:**
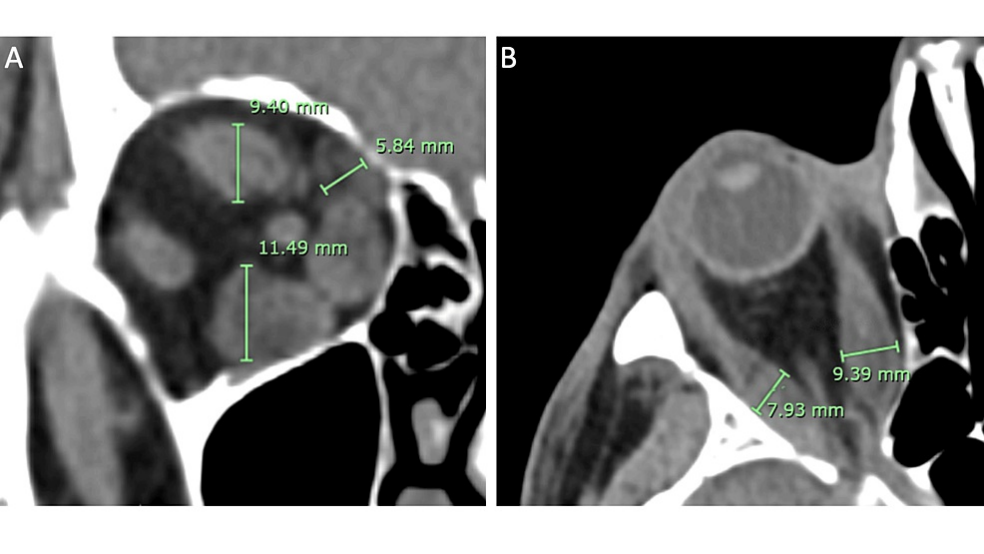
Maximum extraocular muscle diameter measurements of thyroid eye disease orbit in Synapse PACS version 7.3.000 with ruler annotation tool. A) Coronal plane with measurements of superior rectus complex, superior oblique, and inferior rectus muscle maximum diameters displayed. B) Axial plane with measurements of the medial rectus and lateral rectus muscle maximum diameters displayed.

For analysis of maximum EOM diameters, measurements of the SRC, LR, IR, MR, and SO were performed by two independent reviewers (N.M., K.D.) within Synapse PACS version 7.3.000 (Fujifilm, Tokyo, Japan) using the ruler annotation tool. The horizontal maximum diameters of the MR and LR were measured perpendicular to the long axis of the muscle using axial scans (Figure 3). The maximum diameters of the IR, SRC, and SO were measured using coronal scans. The SRC and IR were measured vertically, and the SO inferolaterally. 

Additional demographic and clinical data recorded for study participants include age, sex, ethnicity, smoking status, clinical activity score, presence of strabismus, presence of upper eyelid retraction, presence of diplopia, and presence of compressive optic neuropathy at the time of the scan.

Sample size calculation

The TED group contained 28 orbits, while the control group contained 31 orbits. 

Statistical analysis

Statistical analysis was performed using the SAS statistical package, version 9.4 (SAS Inc., Cary, NC). We tested for equality of variance using Levene’s test and performed independent-sample t-tests to compare soft tissue volumes and maximum EOM diameters between control and TED orbits. Pearson’s correlation coefficient was used to evaluate the linear relationship between EOM maximum diameters and volumes of both control and TED orbits. Statistical significance is defined as p < 0.0055 using the Bonferroni correction.

## Results

Patient characteristics

Table 1 displays relevant demographic and clinical characteristics of the TED study population. Of the 28 TED orbits, 18 (64.3%) were female and 10 (35.7%) were male. The average age of TED patients at the time of the CT scan was 53.9+13.6 years. At the time of imaging, 82.1% had proptosis, 67.9% had upper eyelid retraction, 42.9% had dysmotility, 17.9% had compressive optic neuropathy, and 17.9% had strabismus; 61.9% had clinical activity scores of 0 to two, while 38.1% had clinical activity scores of three and above; and 10.7% were current tobacco users. The clinical characteristics of age (p = 0.382), sex (p = 0.808), race (p = 0.623), smoking status (p = 0.684), body mass index (p = 0.744), clinical activity score (CAS, p = 0.483), and strabismus (p = 0.174) were demonstrated to have no significant correlation with extraorbital muscle volumes.

**Table 1:** Thyroid eye disease patient clinical and demographic characteristics at the time of CT scan

Characteristic	n	Rate (%)
Mean age (years)	53.92+13.56	
Gender		
Female	18	64.29
Male	10	35.71
Race/ethnicity		
White	10	35.71
Asian	7	25.00
Other/unknown	6	21.43
Black/African American	3	10.71
Hispanic	2	7.14
Tobacco usage		
Never	15	53.57
Former	10	35.71
Current	3	10.71
Clinical characteristics		
Proptosis	23	82.14
Upper eyelid retraction	19	67.86
Dysmotility	12	42.86
Strabismus	5	17.86
Compressive optic neuropathy	5	17.86
Clinical activity score 0-2	13	61.90
Clinical activity score > 3	8	38.10

Volumetric analysis

Table 2 displays the EOM and orbital fat volumes for the control and TED orbits. Compared to controls, the mean SRC volume in TED subjects was 2.3 times enlarged, followed by the IR (2.1 times), SO (1.8 times), MR (1.7 times), LR (1.6 times), and IO (1.6 times) (p < 0.01 for all). The average TED orbital fat volume was 1.4 times larger than the control group average (p < 0.001). 

**Table 2:** Average soft tissue volumes of normal orbits and thyroid eye disease (TED) orbits * Indicates statistically significant difference in volumes between TED and normal orbits (p < 0.0001) ^ Indicates statistically significant difference in volumes between TED and normal orbits (p < 0.01) EOM: extraocular muscle

	TED orbits					Normal orbits	
	Average volume (mm^3) or ratio	95% confidence interval	Ratio TED:Normal	TED orbits > 2 st. deviations above normal (%)	TED orbit volume > normal orbit mean volume (%)	Average volume (mm^3) or ratio	95% confidence interval
Superior rectus complex*	1548.34	1282.04 - 1814.63	2.27	53.57	100.00	680.91	571.15 - 790.67
Inferior rectus*	1006.74	796.62 -1216.85	2.05	50.00	85.71	489.9	430.30 - 549.51
Superior oblique*	470.41	405.28 - 535.53	1.82	32.14	89.29	258.97	209.55 - 308.39
Medial rectus*	1051.05	862.73 - 1239.37	1.71	32.14	85.71	615.33	536.84 - 693.82
Lateral rectus*	869.55	726.62 - 1012.48	1.59	35.71	82.14	548.42	486.53 - 610.31
Inferior oblique^	524.29	391.43 - 657.15	1.57	17.86	82.14	334.78	277.57 - 392.00
Total EOM*	5470.37	4621.15 - 6319.58	1.87	50.00	92.86	2928.32	2549.19 - 3307.44
Fat*	14732.18	13323.18 - 16141.17	1.43	50.00	85.71	10277.61	9560.66 - 10994.57
Fat:EOM ratio^	2.98	2.58 - 3.37	0.81	7.14	10.71	3.68	3.44 - 3.92

The soft tissue mean values with their 95% confidence intervals are portrayed in Figure 1. Figure 2 provides a visual comparison of a normal orbit and a TED orbit in ITK-SNAP 3.8.0, showing the enlargement of EOMs that has occurred in the TED orbit. 

Further subanalysis demonstrated that among those with TED and documented upper eyelid retraction (n = 19), 57.9% had SRC enlargement greater than two standard deviations above normal versus 44.4% of patients without, though this difference was not significantly different (p = 0.204).

Maximum diameter analysis 

Table 3 displays the average maximum diameter of the control and TED orbits. Compared to controls, the IR of TED orbits demonstrated the greatest increase in maximum diameter (1.37 times, p < 0.001). This was followed by the MR (1.35 times, p = 0.001), LR (1.34 times, p < 0.001), SRC (1.14 times, p = 0.021), and SO (1.09 times, p = 0.249). 

**Table 3:** Average maximum diameter of normal orbits and thyroid eye disease (TED) orbits * Indicates statistically significant difference in maximum diameters between TED and normal orbits (p < 0.01)

	TED orbits					Normal orbits	
	Average maximum diameter (mm)	95% confidence interval	Ratio TED:Normal	% TED orbits > 2 S.D. above normal (%)	% TED orbits > normal orbit mean maximum diameter (%)	Average maximum diameter (mm)	95% confidence interval
Superior rectus complex	5.68	5.15 - 6.22	1.14	32.14	64.29	4.98	4.80 - 5.17
Inferior rectus	6.08	5.38 - 6.79	1.35	53.57	75.00	4.50	4.32 - 4.68
Superior oblique	2.70	2.35 - 3.05	1.09	17.86	50.00	2.48	2.35 - 2.60
Medial rectus*	5.02	4.45 - 5.59	1.35	35.71	89.29	3.71	3.53 - 3.89
Lateral rectus*	3.68	3.37 - 4.00	1.34	25.00	92.86	2.75	2.54 - 2.96

Table 4 displays the correlation between maximum muscle diameter and volume of control and TED orbits. Among TED orbits, maximum EOM diameters correlated strongly with EOM volumes (IR: r = 0.906, p < 0.001; MR: r = 0.830, p < 0.001; LR: 0.604, p < 0.001; SRC: r = 0.800, p < 0.001; SO: r = 0.518, p = 0.005). Among control orbits, there was little to no correlation between maximum EOM diameters and volumes (IR: r = 0.327, p = 0.073; MR: r = -0.023, p = 0.904; LR: 0.037, p = 0.845; SRC: r = 0.144, p = 0.440; SO: r = -0.262, p = 0.154).

**Table 4:** Correlation between extraocular muscle (EOM) volume and the maximum diameter of normal orbits and thyroid eye disease (TED) orbits

	TED orbits		Control orbits	
	Pearson’s correlation coefficient (r)	p-value	Pearson’s correlation coefficient (r)	p-value
Superior rectus complex	0.800	<0.001	0.144	0.440
Inferior rectus	0.906	<0.001	0.073	0.698
Superior oblique	0.518	0.005	-0.262	0.154
Medial rectus	0.830	<0.001	-0.023	0.904
Lateral rectus	0.604	<0.001	0.037	0.845

## Discussion

Earlier studies from the 1970s to the 1990s have suggested that the most commonly enlarged EOMs in patients with TED are the IR and MR muscles, followed by the SR, LR, SO, and IO muscles [2-4], with only Nugent et al. finding the super rectus and levator palpebrae superioris to be most frequently enlarged [10]. However, the methodologies used to determine the degree of EOM enlargement in these older studies are either purely subjective or less rigorously quantitative. For example, Nugent et al. compared muscle diameters, with only one diameter measured per muscle [10]. Enzmann et al. graded EOM enlargement on CT scans using a subjective scale of 0 to +4 [2]. Wiersinga et al. subjectively graded EOM volumes as definitely increased, dubiously increased, or not increased [3]. Forbes et al. calculated volumes using pixel attenuation on CT scans but only examined a few slices of each scan [4].

While some recent studies have found the IR to be most involved [11, 13], others have begun to challenge the IMSLO pattern of involvement. In a study of 92 orbits in patients with TED-related strabismus, Del Porto et al. found that the mean SO muscle cross-sectional area was greater than three standard deviations above (or >250% larger) the control group mean in 96% of TED orbits [5]. By measuring cross-sectional area, Davies et al. found that in TED patients with upper eyelid retraction, the levator palpebrae superioris muscle was the most frequently enlarged compared to other EOMs [6].

Our study, based on a clinic cohort, supports these studies in suggesting the relatively more common involvement of both the SO and the SRC in TED compared to earlier studies. In our study, the SRC was the most frequently enlarged, with 100% of TED orbits showing some degree of SRC enlargement. It was also the most enlarged in terms of volume, with the mean SRC volume of the TED cohort being 2.3 times larger than that of the normal orbits (Table 2). The second-most enlarged muscle was the IR, with the TED cohort's mean volume being 2.1 times larger than the normal cohort’s.

Additionally, the SO was enlarged to a greater degree than the medial rectus in our TED cohort (1.8 times enlarged vs. 1.7) (Table 2). It was also more frequently enlarged (89.3% enlarged vs. 85.7%). There was a trend of greater SO enlargement among patients with strabismus and/or dysmotility. The 43% with dysmotility had larger SO volumes than those without (2.0 times enlarged with dysmotility versus 1.7 times enlarged without). However, these differences were not statistically significant. 

Upper eyelid retraction is the most common symptom of TED, is present in roughly 80% of patients, and can cause not only cosmetic disfigurement but also lagophthalmos and exposure keratopathy [14,15]. Hypotheses for the mechanism of upper eyelid retraction include the mechanical disruption of the eyelid secondary to proptosis, fibrosis and inflammation of the Muller's muscle, sympathetic overstimulation of the Muller’s muscle, weakened orbicularis muscle leading to decreased levator opposition, and levator palpebrae superioris overaction [6,16]. Davies and Dolman examined 50 patients with unilateral TED with upper eyelid retraction, using the contralateral eye as the control, and found that the levator palpebrae superioris muscle was significantly larger on the side with eyelid retraction [6]. Similarly, another study found that 83% of TED orbits with upper eyelid retraction had enlarged levator palpebrae superioris muscles, versus just 3% of orbits from patients with Graves disease without eyelid retraction [8]. These studies, in addition to Byun et al.’s, posit that enlargement of the levator palpebrae superioris is the major mechanism responsible for upper eyelid retraction [6-8]. However, Byun et al. found no direct relationship between the degree of enlargement and the degree of retraction. In cases where the SRC was not enlarged but the patient nevertheless had upper eyelid retraction, they suggest that, in line with Hering’s law, the SRC is over-contracted in response to contralateral inferior rectus hypertrophy [7].

All TED orbits had some degree of SRC enlargement compared to mean normal values, and 68% of the TED cohort who had upper eyelid retraction had larger mean SRC volumes than those without retraction, though this difference was not statistically significant. As our study combined the superior rectus with the levator palpebrae superioris, we were unable to differentiate which was chiefly responsible for the enlargement. However, the lack of a significant difference in SRC enlargement between orbits with and without upper eyelid retraction is consistent with Byun et al., who found no correlation between SRC volume and the magnitude of upper eyelid retraction [7]. Additionally, in a study of 19 patients with TED with isolated SRC enlargement, only 63% of patients had upper eyelid retraction, supporting the theory that while SRC enlargement likely plays a large role in upper eyelid retraction, the etiology is multifactorial [9]. There are currently no validated management guidelines for upper eyelid retraction caused by TED, though lid-lengthening surgery, hyaluronic acid fillers, botulinum toxin, and local steroids have been used [14,17,18]. Notably, while teprotumumab has been shown to decrease EOM volume, it has not been shown to result in improved eyelid retraction [19,20]. 

As for fat enlargement, our TED orbits had relatively less fat enlargement in comparison to EOM enlargement. In the 50% of the TED cohort with fat volume that was greater than two standard deviations from normal, proptosis, eyelid retraction, and dysmotility (which includes diplopia, strabismus, and EOM restriction) were not significantly different compared to the other 50% of the cohort. Our findings are consistent with the literature. One study found that 61% of TED orbits had increased EOM volume with normal fat volume, while only 5% had increased fat volume with normal EOM volume [16,21]. One study suggests that fat-dominant TED is associated with upper and lower eyelid retraction and proptosis, while EOM-dominant TED is associated with diplopia, strabismus, and EOM restriction [22]. However, our study population and volume measurements do not support those associations.

In addition to our volumetric analysis, we examined the maximum EOM diameters within the same cohorts of TED and normal orbits to determine whether EOM enlargement in TED was consistent across both measurement techniques. As measured by maximum muscle diameter, the IR and MR muscles exhibited the greatest enlargement and the highest frequency of enlargement greater than two standard deviations above normal (Table 3). These findings contrasted with our volumetric analysis and aligned with the traditional understanding of EOM enlargement in TED [1,2,5,6]. These observed differences between measurement techniques may be attributed to physiologic variations in the diameters of EOMs along their lengths and the non-uniform muscle enlargement patterns in TED [23,24].

While we observed a strong positive correlation between the maximum diameters and volumes of EOMs within TED orbits, there was little to no correlation between the two measurement techniques in normal orbits. Szucs-Farkas et al. found a similar disparity between TED and normal orbits when correlating EOM cross-sectional areas and volumes and attributed these findings to differences in the cross-sectional shapes of EOMs in TED and normal orbits [25]. We hypothesize that a similar phenomenon may account for the findings of the present study. The enlarged EOMs of TED orbits tend to be ellipsoid or round in cross-section, while the EOMs of normal orbits are more flat. As such, a single maximum diameter measurement may better correlate with EOM volume in TED orbits. Our findings emphasize that maximum diameter is a less informative measurement overall compared to volume, although as a screening method for detecting TED, it may be quite useful. 

One limitation of our study is the grouping of the SR and levator palpebrae superioris together into the SRC. Ideally, each muscle would be measured and analyzed separately; however, the decision was made to group the superior rectus and levator palpebrae superioris together as they individually were not accurately distinguishable on most CT scans. Another limitation is that only patients with CT scans of sufficient resolution were included in the study, and patients with more severe TED requiring imaging may be overrepresented in our study cohort, as patients with more clinically mild TED are less likely to undergo imaging. Further, being based at a tertiary referral center, our population may reflect relatively more severe TED patients. We were also unable to compare demographic characteristics or age matches as our normal orbit cohort lacked demographic information. Finally, in our cohort of 28 TED orbits, each orbit was manually segmented only once due to the time-intensive nature of manual segmentation, but previous evaluation of our normal orbit manual segmentations showed good interrater correlation (unpublished data).

## Conclusions

Our findings suggest that the SRC, rather than the IR, maybe more frequently and more severely affected with respect to volume enlargement in TED than previously thought. The SO was also more commonly involved and more severely enlarged compared to the LR, contrary to earlier studies.

Our study is unique in that we used volumes rather than diameters or cross-sectional areas to extrapolate EOM volume, as other studies have done. By measuring volume, we are able to more accurately quantify muscle enlargement compared to proxies such as diameter or cross-sectional area measurements. Maximal diameter measurements correlated more strongly with IR and MR volumes and underrepresented the actual volume in the SRC and superior oblique in TED orbits.

## References

[1] Bahn RS (2010). Graves' ophthalmopathy. N Engl J Med.

[2] Enzmann DR, Donaldson SS, Kriss JP (1979). Appearance of Graves' disease on orbital computed tomography. J Comput Assist Tomogr.

[3] Wiersinga WM, Smit T, van der Gaag R, Mourits M, Koornneef L (1989). Clinical presentation of Graves' ophthalmopathy. Ophthalmic Res.

[4] Forbes G, Gorman CA, Brennan MD, Gehring DG, Ilstrup DM, Earnest F 4th (1986). Ophthalmopathy of Graves' disease: computerized volume measurements of the orbital fat and muscle. AJNR Am J Neuroradiol.

[5] Del Porto L, Hinds AM, Raoof N, Barras C, Davagnanam I, Hancox J, Adams G (2019). Superior oblique enlargement in thyroid eye disease. J AAPOS.

[6] Davies MJ, Dolman PJ (2017). Levator muscle enlargement in thyroid eye disease-related upper eyelid retraction. Ophthalmic Plast Reconstr Surg.

[7] Byun JS, Lee JK (2018). Relationships between eyelid position and levator-superior rectus complex and inferior rectus muscle in patients with Graves' orbitopathy with unilateral upper eyelid retraction. Graefes Arch Clin Exp Ophthalmol.

[8] Ohnishi T, Noguchi S, Murakami N (1993). Levator palpebrae superioris muscle: MR evaluation of enlargement as a cause of upper eyelid retraction in Graves disease. Radiology.

[9] Wang Y, Mettu P, Broadbent T (2020). Thyroid eye disease presenting with superior rectus/levator complex enlargement. Orbit.

[10] Nugent RA, Belkin RI, Neigel JM, Rootman J, Robertson WD, Spinelli J, Graeb DA (1990). Graves orbitopathy: correlation of CT and clinical findings. Radiology.

[11] Fidor-Mikita E, Krupski W (2008). Computed tomography imaging of orbits in thyroid orbitopathy. J Pre Clin Clin Res.

[12] Dagi LR, Zoumalan CI, Konrad H, Trokel SL, Kazim M (2011). Correlation between extraocular muscle size and motility restriction in thyroid eye disease. Ophthalmic Plast Reconstr Surg.

[13] Li Q, Ye H, Ding Y (2017). Clinical characteristics of moderate-to-severe thyroid associated ophthalmopathy in 354 Chinese cases. PLoS One.

[14] Chang EL, Rubin PA (2002). Upper and lower eyelid retraction. Int Ophthalmol Clin.

[15] Jain AP, Gellada N, Ugradar S, Kumar A, Kahaly G, Douglas R (2022). Teprotumumab reduces extraocular muscle and orbital fat volume in thyroid eye disease. Br J Ophthalmol.

[16] Dutton JJ (2018). Anatomic considerations in thyroid eye disease. Ophthalmic Plast Reconstr Surg.

[17] Osaki TH, Monteiro LG, Osaki MH (2022). Management of eyelid retraction related to thyroid eye disease. Taiwan J Ophthalmol.

[18] Grisolia AB, Couso RC, Matayoshi S, Douglas RS, Briceño CA (2017). Non-surgical treatment for eyelid retraction in thyroid eye disease (TED). Br J Ophthalmol.

[19] Simmons BA, Tran C, Pham CM, Shriver EM (2022). The effect of teprotumumab on eyelid position in patients with thyroid eye disease. Plast Reconstr Surg Glob Open.

[20] Jain AP, Jaru-Ampornpan P, Douglas RS (2021). Thyroid eye disease: redefining its management-a review. Clin Exp Ophthalmol.

[21] Wiersinga WM, Regensburg NI, Mourits MP (2013). Differential involvement of orbital fat and extraocular muscles in Graves' ophthalmopathy. Eur Thyroid J.

[22] Kim HC, Yoon SW, Lew H (2015). Usefulness of the ratio of orbital fat to total orbit area in mild-to-moderate thyroid-associated ophthalmopathy. Br J Radiol.

[23] Lee JY, Bae K, Park KA, Lyu IJ, Oh SY (2016). Correlation between extraocular muscle size measured by computed tomography and the vertical angle of deviation in thyroid eye disease. PLoS One.

[24] Su Y, Liu X, Fang S (2022). Age-related difference in extraocular muscles and its relation to clinical manifestations in an ethnically homogenous group of patients with Graves' orbitopathy. Graefes Arch Clin Exp Ophthalmol.

[25] Szucs-Farkas Z, Toth J, Balazs E (2002). Using morphologic parameters of extraocular muscles for diagnosis and follow-up of Graves' ophthalmopathy: diameters, areas, or volumes?. AJR Am J Roentgenol.

